# Epstein Barr virus infection in tree shrews alters the composition of gut microbiota and metabolome profile

**DOI:** 10.1186/s12985-023-02147-3

**Published:** 2023-08-08

**Authors:** Wei Xia, Lei Liu, Nan Shi, Chaoyin Zhang, Anzhou Tang, Guangyao He

**Affiliations:** 1 Department of Otorhinolaryngology Head and Neck Surgery, The First Affiliated Hospital of Guangxi Medical University, Nanning, Guangxi China; 2 Key Laboratory of Early Prevention and Treatment for Regional High Frequency Tumor, Gaungxi Medical University, Ministry of Education, Nanning, Guangxi China

**Keywords:** Epstein-Barr virus, Tree shrew, Gut microbiota, Metabolome profile, Primary infection

## Abstract

**Background:**

Epstein-Barr virus (EBV) infection is a major global threat; its manifestations range from the absence of symptoms to multiorgan malignancies and various gastrointestinal diseases. Analyzing the composition and metabolomic profile of gut microbiota during acute EBV infection might be instrumental in understanding and controlling EBV.

**Methods:**

Six tree shrews were inoculated with EBV by intravenous injection. Blood was collected at regular intervals thereafter from the femoral vein to detect EBV and inflammatory biomarker. At the same time, tree shrew faeces were collected for 16 S rRNA gene sequencing and Non-targeted metabolomics analysis.

**Results:**

16 S rRNA gene characterization along with β diversity analysis exhibited remarkable alterations in gut microflora structure with a peak at 7 days post-infection(dpi). Some alterations in the relative richness of bacterial taxon were linked to infectious indicators. Of note, *Butyricicoccus* relative richness was positively linked to EBV presence in the blood and plasma, the opposite correlation was seen with *Variovorax* and *Paramuribaculum*. Non-targeted metabolomics indicated the fecal metabolome profile altered during EBV infection, particularly 7 dpi. The relative abundance of geranic acid and undecylenic acid in stool samples was positively linked to systemic inflammatory biomarkers, and an inverse relationship was reported with the estrone glucuronide, linoleic acid, protoporphyrin IX and tyramine.

**Conclusion:**

Collectively, EBV infection in this model correlated with changes in the composition and metabolome profile of the gut microbiota.

**Supplementary Information:**

The online version contains supplementary material available at 10.1186/s12985-023-02147-3.

## Background

Epstein-Barr virus (EBV) is a ubiquitous gammaherpesvirus [1, 2]. In most humans, persistent EBV infection is asymptomatic; however, in patients with congenital or acquired immunodeficiencies, the loss of immune control can result in EBV-driven proliferation of malignant lymphomas [3]. EBV infection is also associated with the development of malignancies of lymphoid and epithelial origin, including nasopharyngeal carcinoma, gastric carcinoma, Burkitt Lymphoma, and Hodgkin Lymphoma [4, 5]. Importantly, evidence of the association of EBV with various gastrointestinal diseases has also been observed [6–8]. More than 200,000 novel cancer cases are EBV-related, and approximately 140,000 deaths are attributed to EBV-associated malignancies worldwide [9].

Altered gut flora is associated with the pathogenesis of some viral infections [10–14], however, little is known about the effects of EBV infection on the intestinal microflora. A major obstacle is the lack of validated animal models that recapitulate human infection [15, 16]. The Chinese tree shrew (*Tupaia belangeri chinensis*) is a small mammal similar in appearance to squirrels and is genetically closer to primates than rodents [17]. Interestingly, a recent study on the gut microbiota of the ferret, marmoset, woodchuck, mini pig, and tree shrew suggested that the overall distribution of gut microbial species in the gut mirrors their host taxonomic phylogeny [18]. This shows that the tree shrew is suitable for studying intestinal flora. Indeed, it has been reported that the changes in gut microbiota, disease symptoms, and histopathology following *C. difficile* infections in tree shrews are congruent with those observed in humans [19].

In the context of an infection, human data are highly important but do not enable the entire course of infection (i.e., from before contamination until after resolution) to be monitored. Numerous studies have demonstrated that the tree shrew is a practical small animal model for a variety of human virus studies [20–22]. Our previous study demonstrated that tree shrews are a suitable model for EBV infection [23]. This model recapitulates the mild disease that has been observed in the majority of human EBV cases without overt clinical symptoms. In the present study, we monitored changes over the course of infection in gut microbiota composition and metabolome profile. Our results showed that experimental EBV infection alters the gut microbiota in terms of taxonomic composition and, more importantly, metabolome profile.

## Materials and methods

### Animal experiments and ethics Statement

**Fig. 1:**
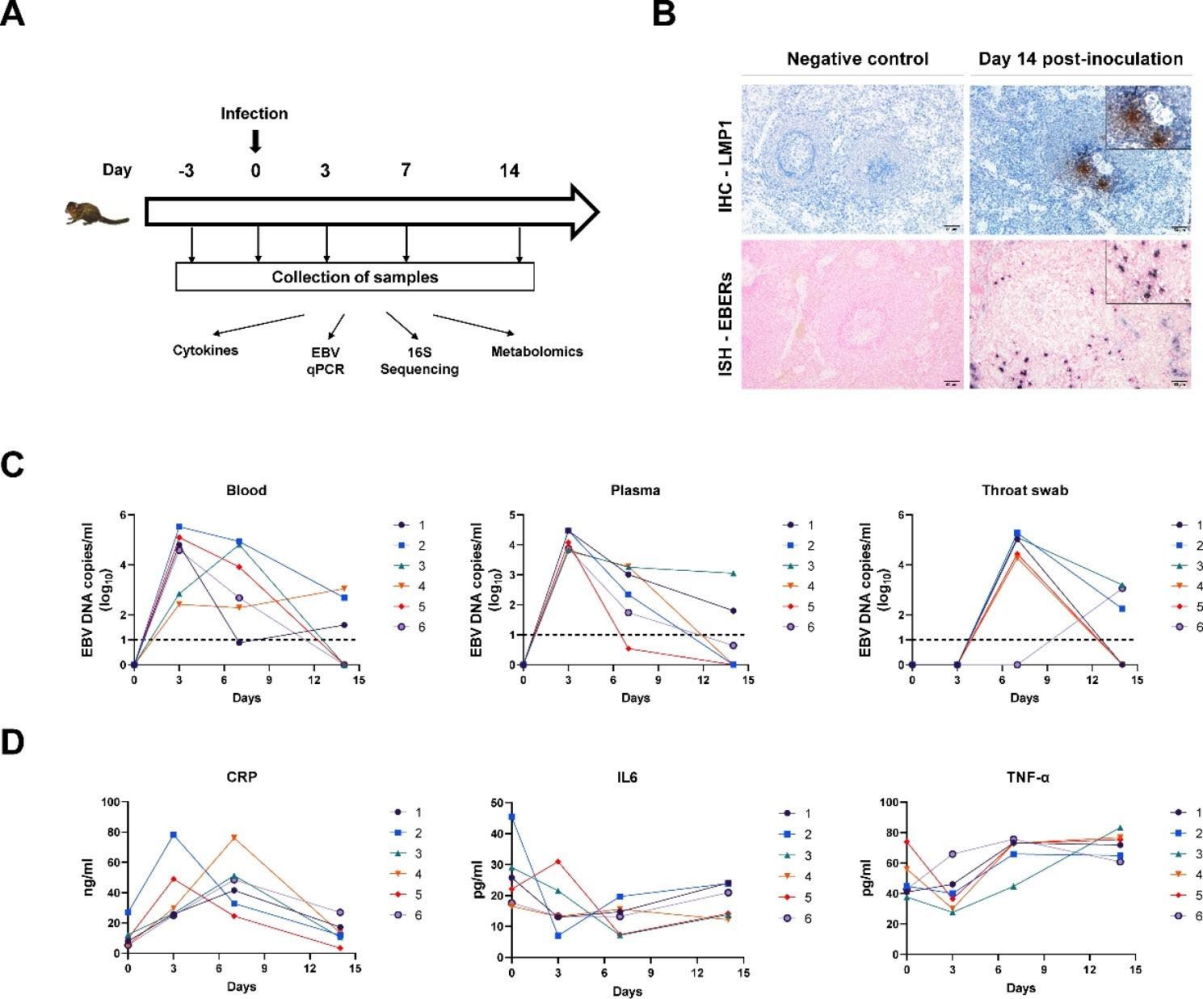
EBV infection of tree shrews. **(A)** Infection of tree shrews by EBV, and collection of samples. Quantifying viral loads and systemic factors was done before infection (day − 3 designates the basal line) and at diverse post-infection time points. **(B)** Immunohistochemical (IHC) detection of LMP1 protein in the spleen at 14 dpi. Positive viral proteins and negative cells are shown in brown and blue, respectively. EBERs expression in spleen, detected by in situ hybridization (ISH) at 14 dpi. Positive staining is gray/black, nuclei counterstained with neutral red. The negative controls sections used for ISH and IHC were from the same infected animals but processed for IHC with no primary antibody, or with no probes for ISH. **(C)** Viral loads (determined by RT-qPCR assay) in blood, plasma, and Throat swab; **(D)** The serum C-reactive protein(CRP), interleukin (IL)-6 and Tumor Necrosis Factor (TNF)-α concentration

Six tree shrews (Production approval number: SCXK (Dian) 2020–0004, 8 ± 6 months, half male and half female, 126.4 ± 6.3 g) were inoculated with 200 µl EBV suspensions(1 × 10^8^ copies/ml) via the femoral vein injection. Blood, throat swab and fecal samples were collected on the 3 dpi, 7 dpi and 14 dpi(femoral vein blood, 1 ml each time). Samples collected at 3 days prior to infection used as control baseline values to determine possible changes following infection(Fig. 1A). The tree shrews were sacrificed at 14 dpi with an overdose of sodium pentobarbital. Subsequently, whole pieces of the spleens were carefully collected and fixed in 4% paraformaldehyde followed by dehydration in serial alcohol dilutions. EBV preparation and other molecular biological experiments were performed as described in a previous publication [23], and full details are provided in Supplementary File 1.

### Gut microbiota analysis

Raw quality reads were processed with the data curation pipeline consisting of the initial elimination of low-quality reads using Qiime2 [28]. The reads were assigned to samples based on barcode matching, followed by trimming barcodes along with the primer sequences. Denoising of the sequences was performed via the DADA2 approach, and thereafter classification of reads was performed with SILVA (Release 132, https://www.arb-silva.de/documentation/release-132/), as well as the NT-16 S reference data resource (Supplementary Table 2) [29]. PCA of the Bray–Curtis distance coupled with Analysis of Similarities (ANOSIM) was performed to explore beta diversity. The number of reported species and Chao1, Shannon, and Simpson indices were computed and employed to characterize alpha diversity. Raw sequence data are available from the SRA databank (www.ncbi.nlm.nih.gov/sra) (accession number: PRJNA784168). We conducted differential analysis using the LEfSe pipeline (Supplementary Table 3) [30]. Correlation networks were created using Spearman’s correlation, and figures were created using Cytoscape (V.3.8.0) (Supplementary Table 4). The connectivity degree of a node is defined as the number of edges of that node.

### Metabolomic analysis of fecal samples

The obtained LC-MS data pre-treatment was performed using the XCMS software [31]. Conversion of raw data files into mzXML format was performed, followed by processing with the XCMS, CAMERA, and metaX toolbox included in R. Identification of each ion was performed via the comprehensive data of retention time along with m/z. We recorded each peak’s intensity and generated a 3D matrix harboring the arbitrarily assigned peak indices (retention time–m/z pairs), names of samples (observations), and ion intensity data (variables). Thereafter, the in-house data were matched with the public data resources. Subsequently, KEGG and HMDB were used to annotate metabolites by matching the specific molecular mass information (m/z) with those from the data resource within a threshold of 10 ppm (Supplementary Fig. 2). MetaX was used to pre-process the peak intensity data. Parameters detected in < 50% of QC samples or 80% of test samples were removed, and then the values of the missing peaks were extrapolated with the k-nearest neighbor algorithm to further improve the data quality. PCA was performed to determine outliers and batch effects based on the preprocessed dataset. The fitting of the QC‐centered robust LOESS signal correction into the QC data was performed with respect to the injection order to minimize the drift of signal intensity over time. In addition, we computed the relative standard deviations of the metabolic features across all QC samples and removed those with standard deviations of > 30%.

The group datasets were normalized using the probabilistic quotient normalization algorithm before analysis. The P value analyzed by Student’s t-tests, was then corrected for multiple tests with an FDR (Benjamini–Hochberg), and was used to select different metabolites. We also conducted supervised PLS‐DA via metaX to variables that discriminate profiling statistical approach to determine more specific differences between groups. A VIP cut-off of 1.0 was set to select important features. Metabolic pathway enrichment analysis was conducted using the Joint Pathway Module in MetaboAnalyst 5.0 (http://www.metaboanalyst.ca/MetaboAnalyst/), based on the data of differential metabolites (log2(FoldChange) > |1.5|) and genes (data were obtained from a separate study with RNA-sequencing of tree shrews’ blood after EBV infection at the same time point, and are available under the accession number PRJNA767811) (Supplementary Tables 5 and 6).

### Statistical analysis

All statistical analyses were performed using GraphPad Prism Software v9.0.0 (GraphPad Software Inc., La Jolla, CA, USA). Where appropriate, a two-tailed non-parametric Mann-Whitney test or Kruskal–Walli’s test with Dunn’s multiple comparison was conducted, with P values < 0.05, signifying statistical significance. Correction of p values was performed with the Benjamini and Hochberg approach to control for the false discovery rate.

## Results

### EBV infection of tree shrews

Six shrews were infected with 1 × 10^8^ copies of EBV isolated from the B95-8 strain (NCBI: txid 10,377) via the femoral vein (Fig. 1A). qPCR was used to determine viral loads in the blood, plasma, and throat swabs. For blood and plasma, the peak level appeared on 3dpi(compared to 0 dpi, 7 dpi or 14 dpi, p < 0.05), while the peak level of throat swabs appeared on 7 dpi(compared to 0 dpi, 3 dpi or 14 dpi, p < 0.05)(Fig. 1C, Supplementary Table 7). Although viral loads decreased markedly on 14dpi, the positive signals of EBV-encoded protein1 (LMP1) and EBV-encoded small RNAs(EBERs) were detected in spleen tissues which implied a latent infection being established(Fig. 1B). Inflammation is typically induced in response to a microbial infection. We observed serum levels of the inflammatory marker C-reactive protein (CRP), interleukin (IL)-6 and Tumor Necrosis Factor (TNF)-α. Serum CRP and TNF-α levels peaked on 7 dpi(compared to 0 dpi or 14 dpi, p < 0.05), while serum IL-6 levels did not show an increase trend **(**Fig. 1D, Supplementary Table 7). Altogether, our results illustrate that tree shrews are susceptible to EBV experimental infection and concomitant inflammatory factor changes in the early phase of virus replication.

### EBV infection alters the structure of the fecal microflora in tree shrews

**Fig. 2:**
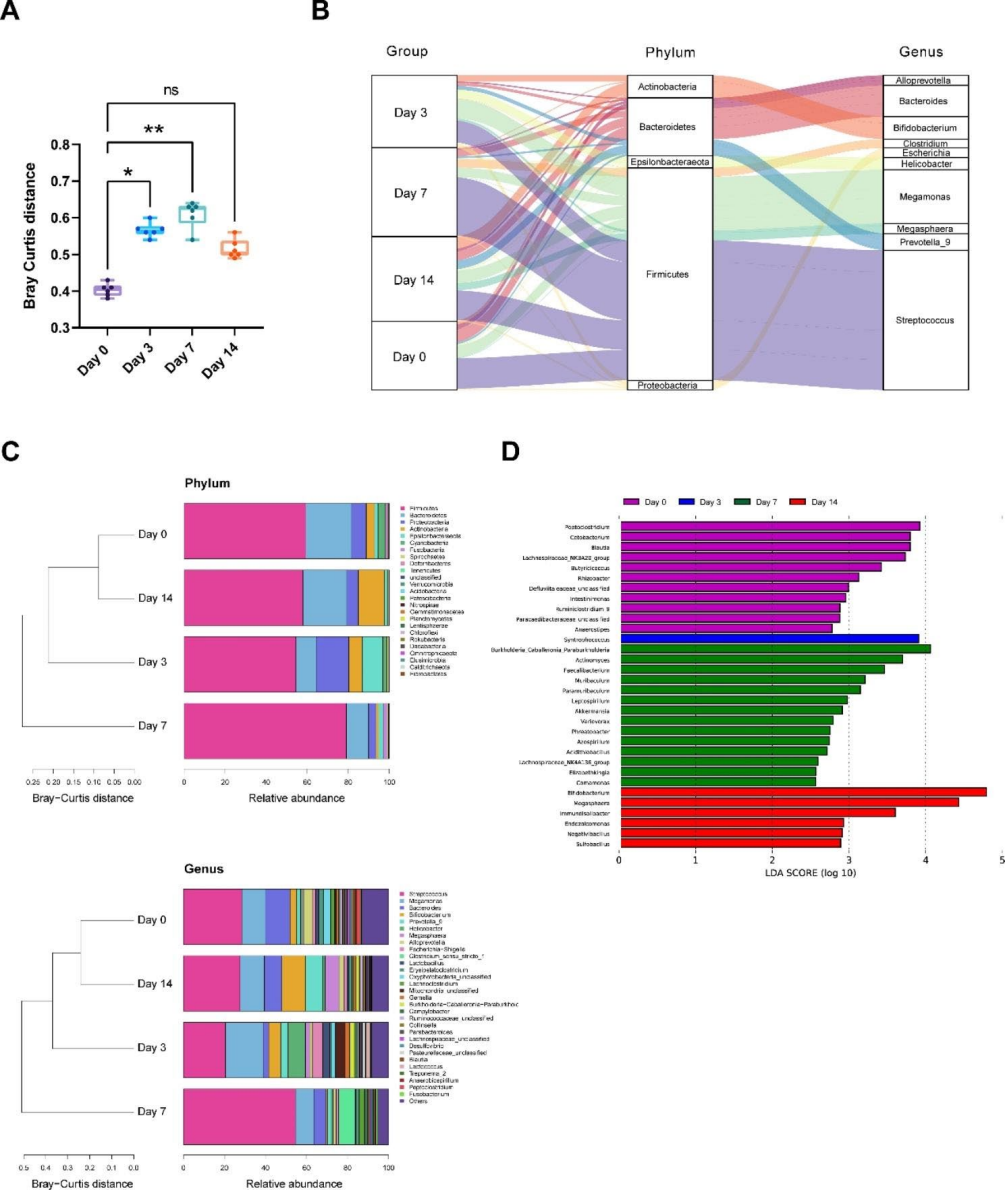
Alterations in the structure of bacterial gut microbiota over time. **(A)** Beta diversity was determined. Bray Curtis distance between specified time point and Day 0. The overall structure of the bacterial microflora **(B)** at the Phylum and Genus **(C)** levels was assessed for each time point during EBV infection. **(D)** A LEfSe assessment exhibits that diverse bacterial taxa changed over the EBV infection. Only taxa with a remarkably significant LDA score (log10) > 1.5 are shown. *p < 0.05, **p < 0.01

The influence of EBV on the structure of the gut microflora was explored. Stool specimens were acquired before and during infection (six duplicate specimens at each time point), and 16 S rRNA sequencing was used to explore the microflora structure. Overall, 1,568,536 sequence reads (mean = 65,356; min = 53,997; max = 79,157; Supplementary Table 1) were obtained. There were no remarkable alterations in alpha diversity with respect to the number of reported species or Shannon indices over the course of infection (Supplementary Fig. 1A and 1B). Beta diversity analysis based on the Bray–Curtis index showed clustering per species and inter-individual variability (Supplementary Fig. 1C). Nonetheless, assessment of the Bray–Curtis distance on day 0 relative to the other time points exhibited a drift in the fecal microflora after infection from 3 dpi and a peak at 7 dpi (Fig. 2A). Notably, β diversity returned to basal levels at 14 dpi post-infection. These data illustrate that EBV infection triggers changes in gut microflora structure.

At the phylum level, *Firmicutes* were the most enriched bacteria, followed by *Bacteroidetes*, *Proteobacteria*, and *Actinobacteria* in the fecal microflora of tree shrews. At genus level, several bacteria(*Streptococcus, Megamonas, Bifidobacterium*) with the highest the relative abundance stood out (Fig. 2B). Visualization of variation in phylum level and genus level relative abundance across sampling time points can be found in Fig. 2C. It is clear that the composition of gut microbiota structure significantly altered during EBV infection. At the same time, the alterations in bacterial taxon richness during infection was further supported by linear discriminant analysis effect size(LEfSe) analysis. Some fecal microflora alterations remained present at 14 dpi, even though they varied from those reported at earlier time points. In particular, the abundance of several *Syntrophococcus* genus members (*Firmicutes* phylum) was higher at earlier time points. Even though a remarkable number of changes were reported at 7 dpi and resolved at later time points, other alterations were reported post-EBV infection at 14 dpi. Altogether, EBV infection in tree shrews was linked to alterations in the composition of the gut microflora, peaking at 7 dpi.

### EBV infection influences the fecal microflora cross talk network

**Fig. 3:**
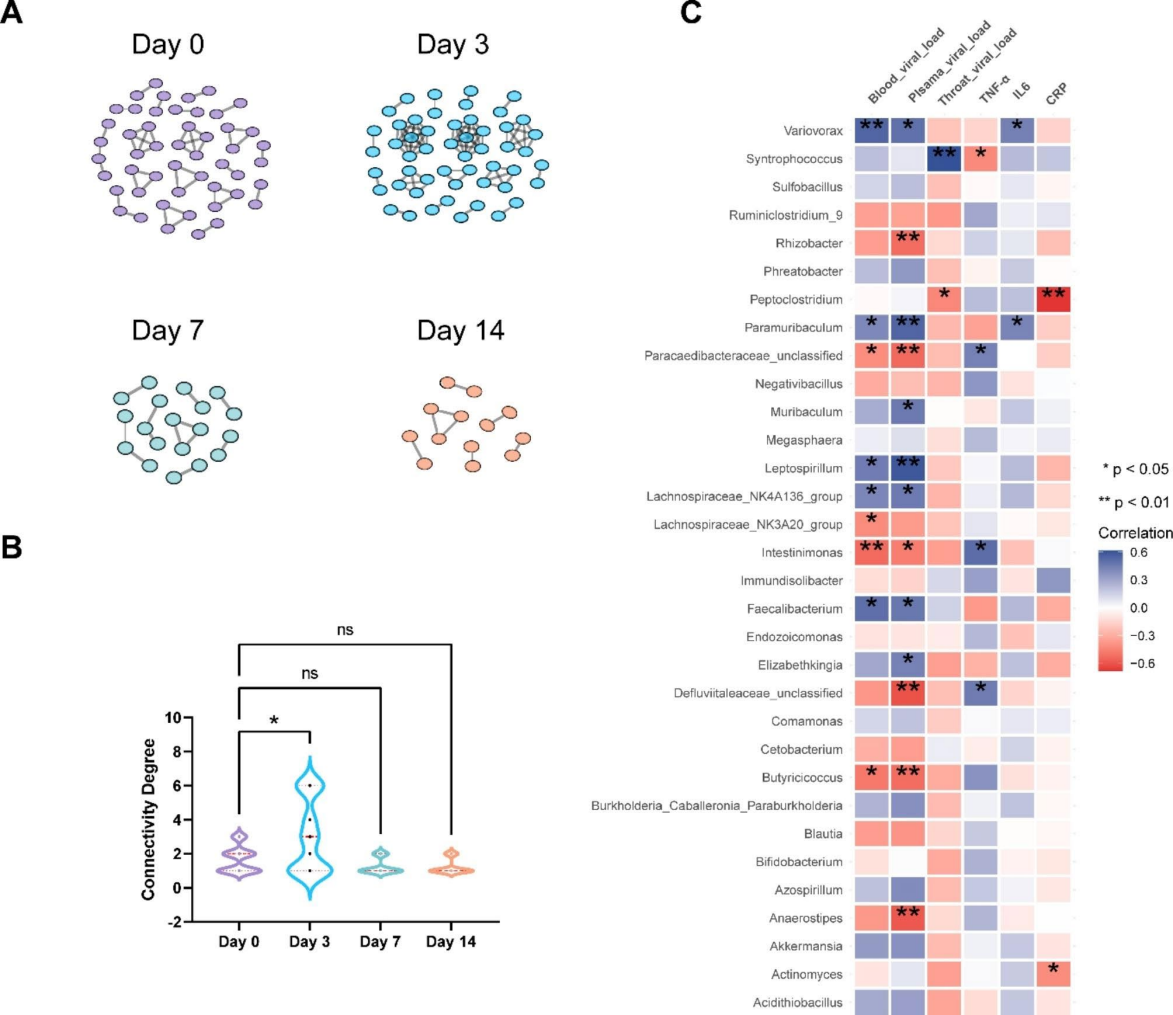
Correlations between the changes in the fecal microflora structure and infection-linked variables. **(A)** Correlation networks at the genus level for bacterial richness were created via Spearman’s correlation approach for four time periods. Each circle (node) designates a genus. **(B)** Violin plots show the degree of connectivity for the four time periods indicated. **(C)** The heat map shows the correlations between the representation of the various bacterial taxa and infection-linked variables. *p < 0.05, **p < 0.01

Gut microflora constitutes an ecosystem of cross-talking microorganisms rather than a simple group of individual microbes. Networks built on the correlation of microbial richness can be employed to assess the complex crosstalk of microbial communities, entailing possible interdependence or competition among taxa [34]. A global exploration of the established network yields remarkable information regarding the ecosystem, with a greater number of nodes and connections, illustrating a remarkable crosstalk at the community level. Therefore, we created bacterial richness correlation networks to explore the structure of the microbial ecosystem during EBV infection. To this end, we provided networks for four time points (Fig. 3A). Dense, inter-linked networks were established on day 0 and 3 dpi. In contrast, the network at 7 and 14 dpi was greatly atrophied, harboring few nodes and connections. These data were verified by a quantitative assessment of the degree of connectivity of the four networks (Fig. 3B). Taken together, these data illustrate that EBV infection triggers ecological alterations in the gut microflora.

### Fecal microflora taxa are linked to the features of EBV infection

Blood inflammatory factors are linked to the severity of virus infection [35, 36] and alterations in the relative richness of operational taxonomic units [37, 38]. Hence, we sought to explore whether alterations in the gut microflora structure are linked to EBV infection parameters, including viral load and inflammatory markers (Fig. 3C). Intriguingly, the relative richness of *Butyricicoccus* genus members positively linked to the presence of EBV in the blood and plasma; opposite correlation was observed for the genera *Variovorax* and *Paramuribaculum*. Only one taxon from the genus *Syntrophococcus* negatively correlated with throat EBV. Moreover, *Syntrophococcus* and *Peptoclostridium* were positively linked to TNF-α and CRP levels, whereas an inverse relationship was observed with *Paramuribaculum*. Additionally, a negative correlation was detected between *Variovorax* and IL6.

### EBV infection alters the fecal metabolic profile

**Fig. 4:**
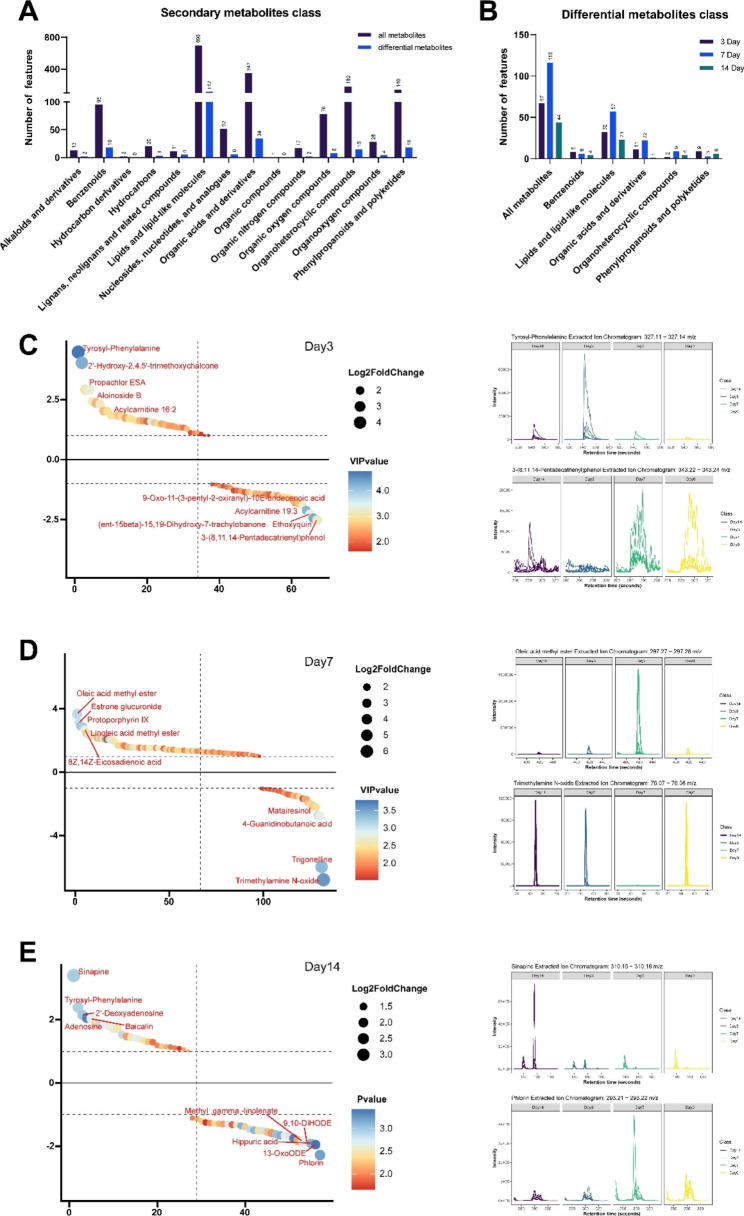
Fecal metabolite generation is altered during an EBV infection. **(A)** Differential secondary metabolites statistics at different classes. Differential metabolites were defined by VIP > 1.5 & p-value < 0.05 compared with the Day 0. **(B)** Differential secondary metabolites statistics at different time points. The figure only shows classes which at least one differential individual per time point. The specific metabolites of the 3 dpi **(C)**, 7 dpi **(D)** and 14 dpi **(E)** are noted, the left panel is the significant differential metabolites, and the right panel is the extracted ion chromatogram of the differential metabolites

To explore the functional influences of infection-linked alterations in fecal microflora structure, we used a non-targeted metabolomics approach to detect metabolites at four time points. To date, no study has examined fecal metabolites following EBV infection. In the present study, 1684 secondary metabolites were identified in the fecal extracts of tree shrews, mainly compounds of the benzenoid class, lipids along with lipid-like molecules class, organic acids coupled with derivative class, organoheterocyclic compound class, phenylpropanoids, and polyketide class (Fig. 4A). Pairwise comparisons of the metabolomic changes in response to EBV infection by OPLS-DA are shown in Supplementary Fig. 3. The 3 dpi, 7 dpi, and 14 dpi clusters could be clearly separated from Day0 in the OPLS-DA model. This suggests that the fecal metabolome profile changed over the course of the infection. As shown in Fig. 4B, compared to Day0 after 7 dpi, EBV infection resulted in the most metabolites that were significantly affected, followed by 3 and 14 dpi. Specifically, there were 67, 116, and 44 differentially expressed metabolites(VIP > 1.5 and p < 0.05) at 3, 7, and 14 dpi, respectively. Most of the significantly differentially expressed metabolites can be classified into lipids, lipid-like molecules, organic acids, and derivatives. The specific metabolites at 3, 7, and 14 dpi are noted in Fig. 4C, D and E; the left panel shows the significant differential metabolites, while the right one shows the extracted ion chromatogram of the differential metabolites. These variations in the fecal metabolome profile might reflect alterations in their generation by bacteria and/or utilization by host cells.

**Fig. 5:**
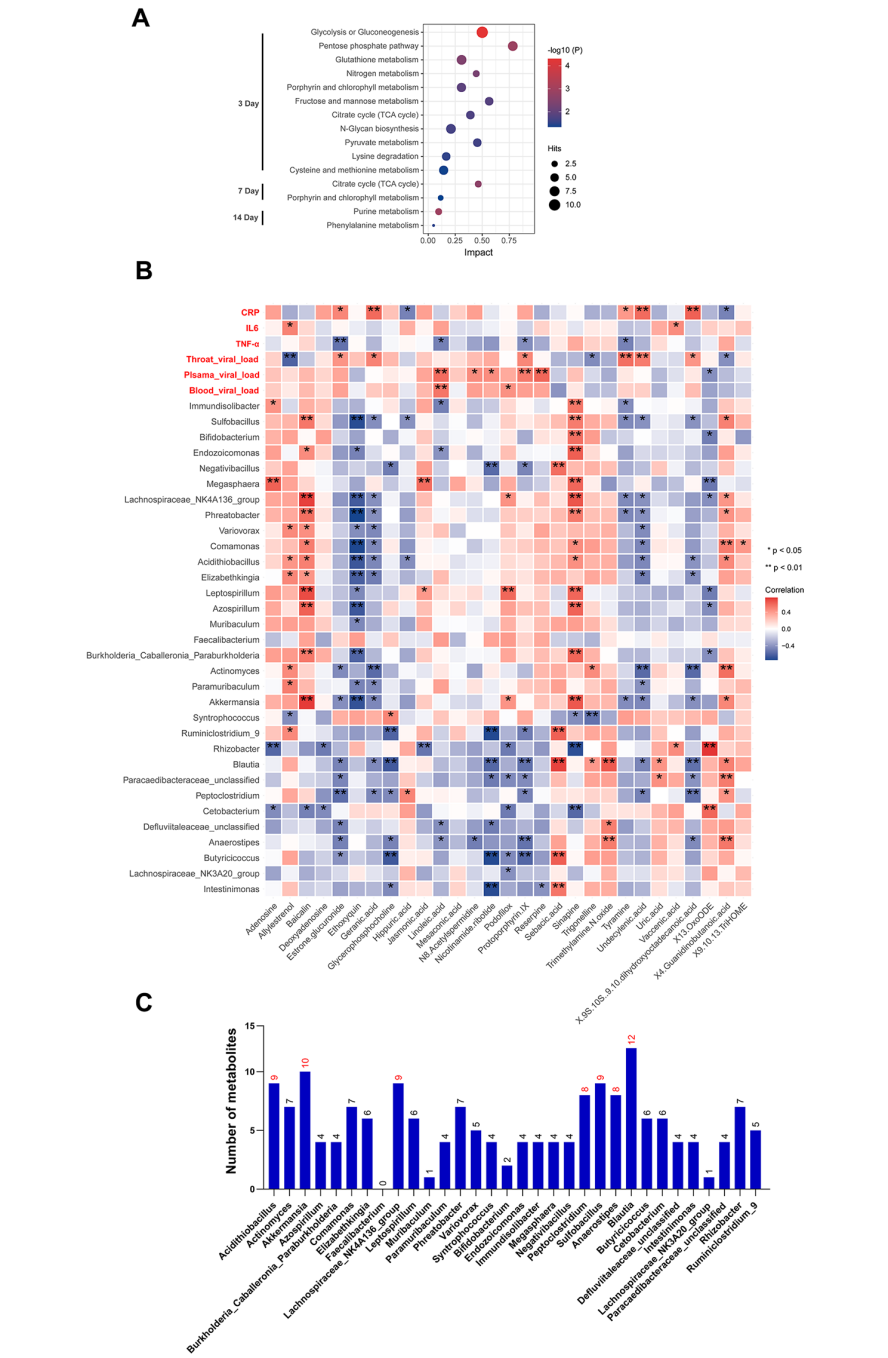
Correlations between the alterations in the fecal metabolites profile and infection-related variables. **(A)** The enrichment results of the KEGG pathway from the KEGG database. **(B)** Relationship of differential fecal metabolite levels with plasma cytokine levels and the representation of the diverse bacterial taxa. *p < 0.05, **p < 0.01. **(C)** The number of metabolites with significant bacterial taxa correlation

The biological pathways affected at different time points were determined based on differentially expressed metabolites and genes. As shown in Fig. 5A, metabolic pathways were significantly enriched at all three time points, while the impact values of pathways decreased over time, until less than 0.1 at 14 dpi. Moreover, the citrate cycle (TCA cycle) and porphyrin and chlorophyll metabolism pathways were affected at both 3 and 7 dpi, indicating that they may serve as possible target pathways for EBV infection.

### Fecal metabolites are linked to the fecal microflora taxa

The correlation between metabolites and infectious parameters indicated that differential metabolites were linked to some infectious parameters (Fig. 5B). Specifically, linoleic acid concentration in stool samples was positively linked to the presence of EBV in the blood and plasma. In contrast, tyramine concentration positively correlated with throat EBV load. The concentrations of estrone glucuronide, linoleic acid, protoporphyrin IX and tyramine negatively correlated with TNF-α levels. Moreover, geranic acid, tyramine, undecylenic acid, and estrone glucuronide positively correlated with CRP levels, while the opposite correlation was observed with hippuric acid and guanidinobutanoic acid. We subsequently analyzed the relationship between gut microbiota and metabolites. The microflora that affected most metabolites were the genera *Blautia*, *Akkermansia*, *Acidithiobacillus*, *Lachnospiraceae_NK4A136_group*, *Peptoclostridium*, *Sulfobacillus*, and *Anaerostipes*, especially *Blautia* (Fig. 5B and C). Interestingly, protoporphyrin IX, enriched in the porphyrin and chlorophyll metabolism pathway, was negatively related to multiple gut microbiota, including the genera *Butyricicoccus* and *Anaerostipes*. Baicalin concentration in stool samples positively correlated with the levels of *Akkermansia* and *LachnospiraceaeNK4A136group* (r > 0.7) and moderately correlated with the levels of *Variovorax* and *Endozoicomonas*. Altogether, these data illustrate those alterations in gut microflora structure during EBV infection are linked to changes in the fecal metabolic profile.

## Discussion

EBV infection poses a threat to public health and the global economy. EBV manifestations range from asymptomatic to multi-organ malignancies and various gastrointestinal diseases [6–8]. Hence, it is pivotal to explore the influence of EBV infection on the gut microflora, as well as the core roles of the latter in human health. Gut microflora alterations during various viral infections have been widely explored in mouse models [12, 39–41]. Still, the influence of experimental EBV infection on the gut microflora remains unknown because rodents do not tolerate EBV infection and replication. In our previous studies, we took advantage of an emerging experimental animal, phylogenetically closer to humans than rodents, and established an EBV infection model [23]. In this study, we evaluated the influence of EBV infection on the structure and the metabolic output of the gut microflora. The application of experimental models to explore the influence of infection on the gut microflora has numerous advantages over human samples. First, the time points of specimen collection (pre-infection, infection, and post-infection) were clearly defined. Second, diverse samples can be collected regularly while complying with ethical rules.

We found that EBV infection triggers alterations in the gut microflora structure, and the peak occurs earlier in infection. This is consistent with viral infections, such as respiratory infections and influenza [10, 11, 42, 43]. These changes occur within a week of infection, and therefore detecting the virus at day 14 does not exactly capture the time point at which viremia occurs and the changes in the microflora take place. Intriguingly, some alterations are linked to viral load and systemic cytokine levels. For example, the richness of the genus *Butyricicoccus* positively linked to the presence of EBV in the blood and plasma, whereas one species from the genus *Syntrophococcus* negatively correlated with throat EBV. Moreover, *Syntrophococcus* positively associated with virus-elicited cytokine levels. This finding is congruent with human data on the relationship between gut microflora, multiple viral infections, and systemic inflammatory markers [14, 44–47]. The present study is the first to document that EBV infection affects fecal metabolome profile. The fecal metabolome profile was altered during EBV infection, particularly at 7 dpi. Nonetheless, it remains unclear whether this alteration influences gut homeostasis (e.g., inflammation and barrier properties) and secondary systemic outcomes; hence, further investigation is required. We observed that the concentration of linoleic acid in stool samples was positively linked to the presence of EBV in the blood and plasma, while that of tyramine was positively linked to the throat EBV load. Moreover, the concentrations of geranic acid and undecylenic acid in stool samples positively correlated with CRP levels, while those of estrone glucuronide, linoleic acid, protoporphyrin IX and tyramine negatively correlated with TNF-α levels. This suggests that changes in the fecal metabolome profile may be involved in the inflammatory process of EBV infection. We also observed a correlation between gut microbiota and metabolites. Our data illustrated that alterations in the gut microflora structure during EBV infection are linked to alterations in the fecal metabolic profile.

The present study is the first to provide evidence of alterations in gut microflora along with the metabolic profile during experimental EBV infection. In contrast with other virus infections in mouse models [13, 48, 49], alterations in the gut microflora communities were less remarkable, and most were reported at low taxonomic levels. This could be attributed to the low pathogenicity of EBV in the tree shrews as long as human EBV infection [4, 50–52]. Indeed, we did not observe gross alteration in fecal consistency and diarrhea or other GI disturbances and no EBV signal(EBERs) was found in formalin-fixed paraffin-embedded tissue samples from EBV infected tree shrew colon and stomach by in situ hybridization. It is worth noting that this limitation is based on correlation analysis. Although it constituted numerous variables (relative richness of numerous taxa at diverse levels), independent biomarkers of EBV infection were not explored. Although our data yield insights for further research, the relationship between bacterial taxa and disease signatures should be investigated. The establishment of appropriate models for EBV is the key to these studies. If clinical and immunological abnormalities triggered by EBV infection is mediated by the gut microflora, targeting the latter’s diverse components could be a possible treatment approach.

### Electronic supplementary material

Below is the link to the electronic supplementary material.

Supplementary Material 1

Supplementary Material 2

Supplementary Material 3

Supplementary Material 4

Supplementary Material 5

Supplementary Material 6

Supplementary Material 7

Supplementary Material 8

Supplementary Material 9

Supplementary Material 10

Supplementary Material 11

Supplementary Material 12

## Data Availability

Raw data have been deposited at the SRA databank (www.ncbi.nlm.nih.gov/sra) and are available under the accession number PRJNA784168.
