# Supplementary figures and images for: Epstein Barr virus infection in tree shrews alters the composition of gut microbiota and metabolome profile
Source: Virol J. 2023 Aug 8;20:177. doi: 10.1186/s12985-023-02147-3 (PMC10410904; doi:10.1186/s12985-023-02147-3)

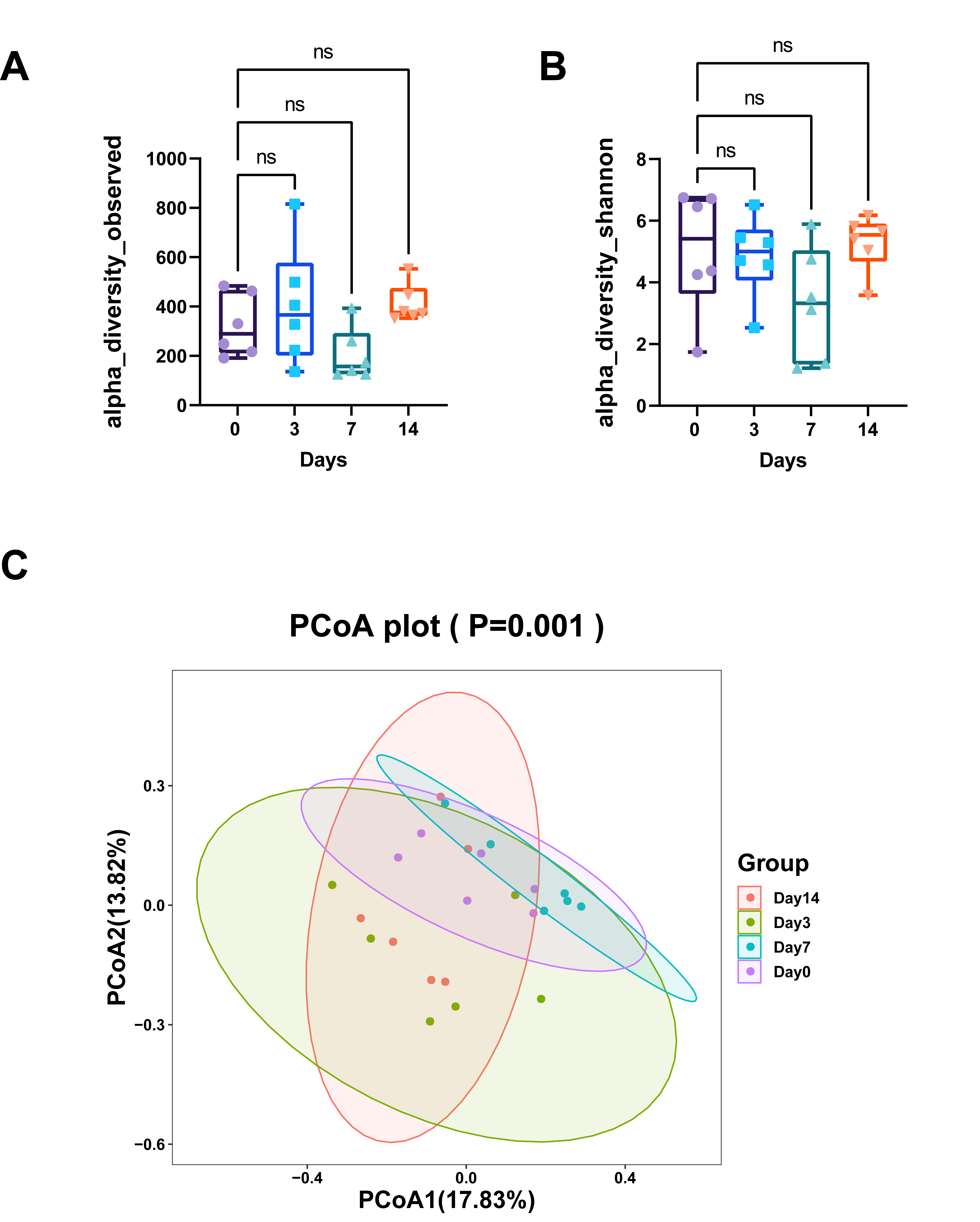

Supplement: Supplementary file 1 — Supplementary Material 1 [file 12985_2023_2147_MOESM1_ESM.tif]

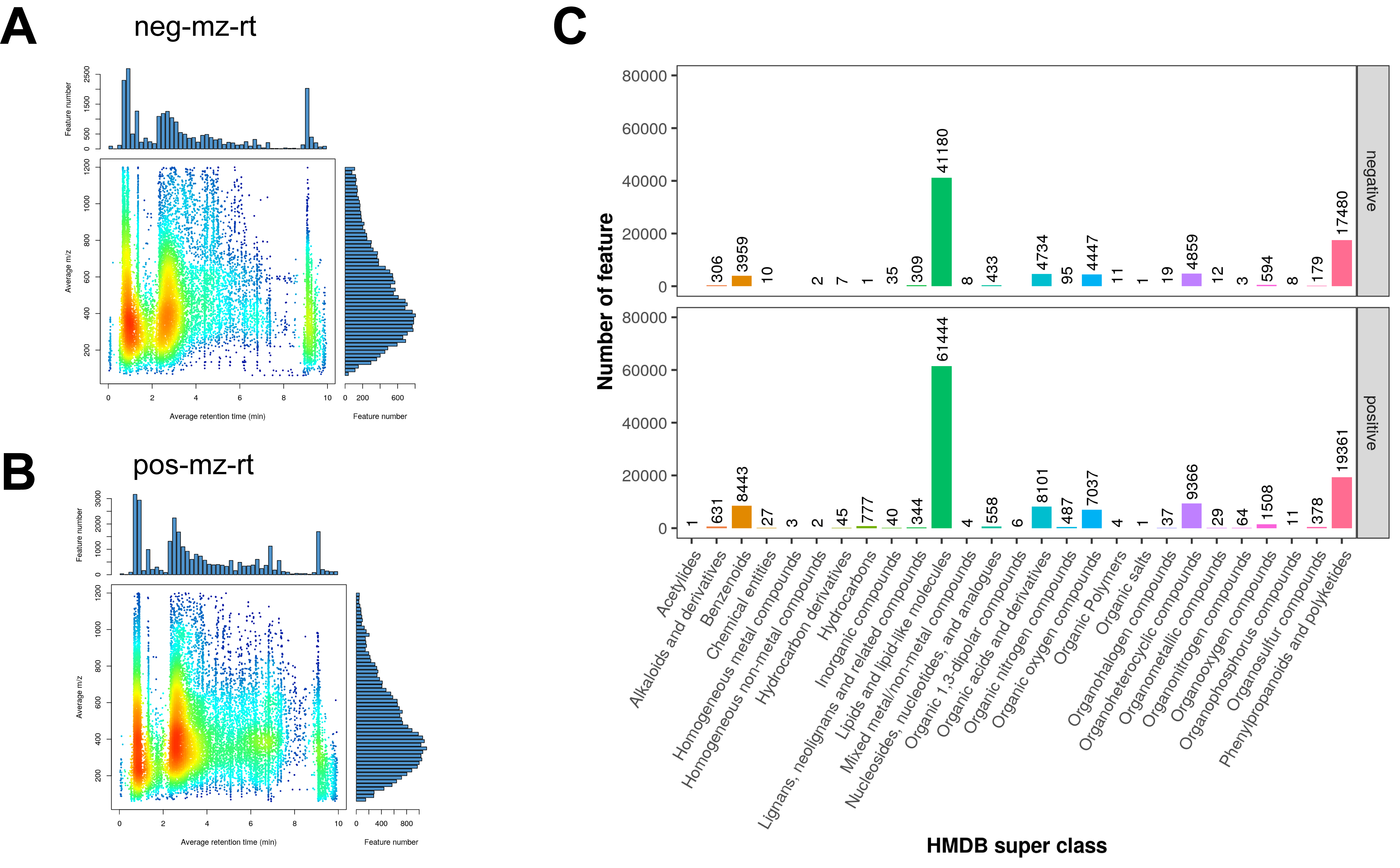

Supplement: Supplementary file 2 — Supplementary Material 2 [file 12985_2023_2147_MOESM2_ESM.tif]
