# Supplementary material for: Epstein Barr virus infection in tree shrews alters the composition of gut microbiota and metabolome profile
Source: Virol J. 2023 Aug 8;20:177. doi: 10.1186/s12985-023-02147-3 (PMC10410904; doi:10.1186/s12985-023-02147-3)
Supplement: Supplementary file 10 — Supplementary Material 10 [file 12985_2023_2147_MOESM10_ESM.pdf]

# 动物实验伦理审查表
# The Tab of Animal Experimental Ethical Inspection

编号(No):202005018

<table>
<tr><td rowspan="10">申请人填写的相关信息(Concerned information written by applicant)</td><td colspan="3">申请人(Applicant)：Xia Wei</td></tr>
<tr><td colspan="3">申请人学历 PHD candidate (Education of applicant)： 技术职称 (Professional title)：</td></tr>
<tr><td colspan="3">实验名称(Study title)：Epstein Barr virus infection in tree shrews alters the composition and functional activity of the gut microbiota</td></tr>
<tr><td colspan="3">实验目的(Aim of experiment)：Using tree shrew model to study the changes of composition and functional activity of the gut microbiota after primary Epstein-Barr virus infection</td></tr>
<tr><td colspan="3">基金来源（Fund sources）This study was supported by the National Natural Science Foundation of China (Grant No. 81760188 ) and the Innovation Project of Guangxi Graduate Education (Grant No. YCBZ2020050) and the National Natural Science Foundation of China (Grant No. 81760189 ) and College Student's Innovation and Entrepreneurship Training Program (Grant No. 201910598142)</td></tr>
<tr><td rowspan="4">拟进动物情况</td><td colspan="2">动物来源(Source of animal)：the Kunming zoology institute, Chinese Academy of Science</td></tr>
<tr><td colspan="2">品种品系(Species or strain)：Tupaia belangeri chinensis 等级(Grade)：cleaning grade 规格(Specifications)：</td></tr>
<tr><td>数量(Number)： 10 只(♀5 只；♂5 只)</td><td>申请日期(Application date)：2020 年 5 月 6 日</td></tr>
<tr><td>进驻日期(Entering date)： 2020 年 6 月 6 日</td><td>结束日期(Ending date)：2021 年 10 月 1 日</td></tr>
<tr><td colspan="3">1.实验要点，实验方法、观测指标<br>Outline of experiments； experimental methods；observational index：<br>2. Inoculate tree shrews with Epstein-Barr virus, check rectal temperature and weight at different time points, and take blood for routine blood tests and other tests. Finally, the animals were sacrificed at the end of the experiment to obtain tissues.<br>3. 仁慈终点或实验终点：<br>Human endpoint or experimental terminative indicator<br>In the process of animal experiment, select the earlier stage of the animal showing pain and depression as the end of the experiment.<br>3.实验结束后处死动物的方法：<br>Executing animal method：<br>Death by overdose of 2% sodium pentobarbital at the end of the experiment<br>4.动物替代、减少动物用量、降低动物痛苦伤害的主要措施等：<br>Major measure for 3Rs：<br>Control group tissues used in the experiment were fresh cadavers that had been euthanized in another study by our research group.</td></tr>
<tr><td></td><td colspan="3">申请人签名(Signature of applicant)：夏魏 联系电话(Telephone)：15978187863</td></tr>
<tr><td>审查结果(是否同意</td><td colspan="3">课题负责人意见 (Study director)： 同意 (Agree) ☑ 不同意 (Disagree) □ 签名 (Signature) [signature]</td></tr>
</table>

<table>
<tr><td rowspan="2">申请人的实验方案)(Results of inspection)</td><td>实验动物设施意见(Opinion from laboratory animal facility):<br><br>同意(Agree) ☑ 不同意(Disagree) □<br><br>签名(Signature) [illegible]</td></tr>
<tr><td>实验动物福利与伦理委员会意见(The Animal Care & Welfare Committee):<br><br>同意(Agree) ☑ 不同意(Disagree) □<br><br>签章(Stamp)</td></tr>
<tr><td colspan="2">备注(Supplement):<br>初审 □ First trial 第 次审查 reexamine No.</td></tr>
</table>

Notes : Animal ethics review follows the *Guiding Opinions on the Treatment of Laboratory Animals* issued by the Ministry of Science and Technology of the People's Republic of China and the *Laboratory Animal-Guideline for Ethical Review of Animal Welfare* issued by the National Standard GB/T35892-2018 of the People's Republic of China. If there are any uncertainties, please refer to these two documents or consult The Animal Care & Welfare Committee of Guangxi Medical University.
