# Supplementary material for: Epstein Barr virus infection in tree shrews alters the composition of gut microbiota and metabolome profile
Source: Virol J. 2023 Aug 8;20:177. doi: 10.1186/s12985-023-02147-3 (PMC10410904; doi:10.1186/s12985-023-02147-3)
Supplement: Supplementary file 11 — Supplementary Material 11 [file 12985_2023_2147_MOESM11_ESM.pdf]

Material and Methods

EBV preparation

The EBV viral supernatant was derived from the B95-8 strain (NCBI: txid 10377), which releases high titers of EBV. All infectious EBV experiments were conducted at biosafety level 2 (BSL2) facilities. The B95–8 strain was cultured in 1640 medium containing 10% FBS and 1×antibiotics (100 kU/L penicillin, 100 mg/L streptomycin) at 37 °C and 5% CO_2 in a humidified incubator for 14 days. After that, the whole culture was transferred to a 50 mL microfuge tube and freeze-thawed three times to lyse cells and release the virus. The culture was then centrifuged at a low speed (1200×*g*) to eliminate cell debris, and the supernatant was further filtered with a 0.45-μm centrifugal filter device. The filtered fluid then underwent high speed spinning at 16,000 × *g* for 90 minutes at 4 °C and resuspended in 1 mL of 1640 medium to obtain the viral supernatant for the experiment. The viral supernatant kept at −80 °C until tree shrew infection [24]. DNA was extracted from 200 μL viral supernatant to determine viral genome copy numbers before use.

Animal Experiments and Ethics Statement

A total of six tree shrews (F1 generation, Production approval number: SCXK (Dian) 2020–0004, 8±6 months, male and female unlimited, 126.4 ±6.3 g) were obtained from the Kunming Institute of Zoology, Chinese Academy of Sciences. We used single cages (35 cm long × 25 cm wide × 30 cm high) to rear the tree shrews. A sleeping rest room

was fabricated in every cage (15 cm long × 12 cm wide ×12 cm high). We reared the tree shrews in moderated temperature (23～25°C), 40～50% relative humidity, as well as 12 hour light/dark cycles. The Animal Ethics Review Committee of Guangxi Medical University granted approval of this research work (approval number: 202005018). The study protocols were as per the 'Guiding Principles for the Use and Care of Experimental Animals' issued by the Ministry of Science and Technology of China and the total number of animals and we minimized their suffering, as per the 3R principle.

Six tree shrews were inoculated with 200μl EBV suspensions($1x10^8$ copies/ml) described previously via the femoral vein injection. Samples were taken the 3 days before inoculation as control. Blood and throat swab samples were collected on the 3 dpi, 7 dpi and 14 dpi after EBV infection (femoral vein blood, 1 ml each time). Plasma and serum were immediately separated through centrifugation at 3,000 g at 4°C and stored at −80 °C. 150 ul of blood samples were used to extract genomic DNA by the QIAamp DNeasy Blood & Tissue kit(Qiagen, Germany) and eluted in 30μl of nuclease-free water. 150μl of tree shrew plasma were used to extract Viral nucleic acid by the QIAamp MinElute Virus Spin Kit and eluted in 20μl of nuclease-free water. Genomic DNA of throat swab samples were extracted using TIANamp Swab DNA Kit(Tiangen, China). The quantity along with the purity of the extracted nucleic acid were determined using a NanoDrop2000 instrument (Thermo Fisher Scientific, USA). And the serum samples were used to detect the levels of CRP, IL6 and TNF-α through ELISA kit (Cusabio, Wuhan, China) following manufacturer guidelines. To study the EBV

infection's influence on the gut microflora, fecal specimen from each challenged tree shrew were sampled 3 days prior to infection, to characterize baseline microbiota composition, and at 3-, 7-, and 14-days post infection. Samples collected at day -3 served as references to determine possible changes in microflora structure following infection. We kept the fecal specimens at −80°C until further analysis.

Quantitative real-time PCR (qPCR) for the detection of EBV copy number

EBV DNA copies of viral supernatant, blood, plasm and throat swab samples were quantified by qPCR, using primers and a probe that target the EBV BamHI W region(forward primer, 5′- TCT TAG GAG CTG TCC GAG GG -3′;reverse primer 5′- CCC AAC ACT CCA CCA CAC C -3′; probe, 5′- CAC ACA CTA CAC ACA CCC ACC CGT CTC -3′) [25]. Standard curves were generated from genomic DNA of Namalwa cell line [26, 27] purchased from the Chinese Academy of Sciences Institute of Cell Resource Center (Shanghai, China), and the culture parameters for the Namalwa cells, as well as the DNA isolation approach, were as documented previously. The PCR reagents used were from Takara Premix ExTaq™ (Probe qPCR), and used per manufacturer-recommended reaction conditions, and all experiments were repeated three times. The viral load of the samples was determined from the appropriate standard curve (PCR amplification efficiency (E) of 95–105% and correlation coefficient (R^2) >0.995) using the Bio-Rad CFX96 detection system.

EBER-in situ hybridization (ISH) for EBV detection in tree shrew spleen tissues

Highly sensitive digoxigenin-labeled oligonucleotide probes were synthesized by Sangon Biotechnology Co., Ltd. (Shanghai, China). Briefly, tissue sections (5 μm) were dewaxed regularly and digested for 30 min with pepsin to expose the RNA. Pre-hybridization was performed for 2–4 hours in a wet box with an aqueous solution of 20% glycerin to reduce nonspecific reactions. The working concentration of the probe was 4 μg/mL. The denaturation step was performed at 95 °C for 15 min. Subsequently, hybridization was performed using an Enhanced Sensitive ISH Detection kit (BOSTER, Wuhan, China). Nuclear Fast Red (Solaibao, Beijing, China) served as a chromogen.

Immunohistochemical (IHC) examination of tree shrews

Spleen tissue samples were prepared and subjected to immunohistochemical analysis using SP-HRP kits (SP-9000, ZSGB Biotechnology Co. Ltd., Beijing, China) following the manufacturer’s instructions. Mouse anti-LMP1 monoclonal antibody was purchased from Abcam Inc(clone number: CS 1-4).

Genomic DNA extraction and sequencing

The DNA E.Z.N.A.®Stool Sample Kit (D4015, Omega, Inc., United States) was used to isolate DNA, as described by the manufacturer. The reagent designed for DNA recovery from trace specimen amounts has been reported to be efficient for isolating most bacterial DNA. A blank was prepared using nuclease-free water. We eluted the DNA in 50 μL elution buffer and kept it at -80 °C for downstream processing. PCR was performed by Outdo Biotech Co., Ltd., Shanghai Province, China. The primers used were tagged with distinct barcodes for every sample, along with sequencing universal

primers at their 5’-ends. A 25 μL PCR reaction volume was set up consisting of the template (25 ng), PCR Premix (12.5 μL), each primer (2.5 μL), and nuclease-free water. The PCR conditions were 98 °C of denaturation for 30 seconds; 32 cycles at 98 °C denaturation for 10s, 54 °C annealing for 30 s, and 72 °C extension for 45 s, and then 72 °C extension for 10 min. The PCR amplicons were confirmed on a 2% agarose gel. Throughout DNA isolation, ultrapure water was used in place of the sample solution as a negative control to eliminate the possibility of false-positive PCR results. We utilized AM Pure XT beads to purify the PCR amplicons (Beckman Coulter Genomics, United States), followed by quantification on a Qubit (Invitrogen, United States). Next, we pooled the amplicon pools, and size selection and quantification were performed using the Agilent 2100 Bioanalyzer (Agilent, United States) and the Illumina Library Quantification Kit (Kapa Biosciences, United States), respectively. Sequencing was performed using the NovaSeq PE250 platform.
